# Assessment of immigration law enforcement presence in a teaching hospital along the US/Mexico border

**DOI:** 10.1186/s12939-023-01934-2

**Published:** 2023-09-28

**Authors:** Claire Lamneck, Alexander Alvarez, Cazandra Zaragoza, Rombod Rahimian, Mario Jesus Trejo, Patricia Lebensohn

**Affiliations:** 1 University of Arizona College of Medicine, Tucson, AZ USA; 2 University of Arizona Mel & Enid Zuckerman College of Public Health, Tucson, AZ USA

**Keywords:** Migrant health, Asylum-seeker, Law enforcement custody, Privacy, Autonomy

## Abstract

**Background:**

Over the past decade, the United States (US) has seen a spike in migration across the US-Mexico border with an increase in hospital admissions of migrants and asylum-seekers under the custody of immigration law enforcement (ILE). This study aimed to determine how the presence of ILE officials affects patient care and provider experience in a teaching hospital setting.

**Methods:**

This cross-sectional online survey solicited quantitative and qualitative feedback from medical students, residents, and attending physicians (n = 1364) at a teaching hospital system with two campuses in Arizona. The survey included participant demographics and addressed participants’ experience caring for patients in ILE custody, including the perception of respect, violations of patients’ privacy and autonomy, and the comfort level with understanding hospital policies and patient rights. Thematic analyses were also performed based on respondent comments.

**Results:**

332 individuals (24%) responded to the survey. Quantitative analyses revealed that 14% of participants described disrespectful behaviors of ILE officials, mainly toward detained patients. Qualitative thematic analyses of respondent comments revealed details on such disrespectful encounters including ILE officers violating the Health Insurance Portability and Accountability Act (HIPAA) and using intimidation tactics with patients. Nearly half of the respondents did not have knowledge of policies about ILE detainees’ medical care, detainees’ privacy rights, or ILE’s authority in patient care.

**Conclusions:**

This study points out the complexities, challenges, and ethical considerations of caring for patients in ILE custody in the hospital setting and the need to educate healthcare professionals on both patient and provider rights. It describes the lived experiences and difficulties that providers on the border face in trying to achieve equity in the care they provide to detained migrant patients.

**Supplementary Information:**

The online version contains supplementary material available at 10.1186/s12939-023-01934-2.

## Background

### Motivation

Over the past decade, the United States (US) has seen a spike in the number of individuals migrating across the US-Mexico border. During the years that this study took place, US Customs and Border Protection (CBP) reported apprehensions of 851,508 migrants in the Southwest Border Sector in fiscal year (FY) 2019 (compared to 396,579 in FY 2018) [1], and US Citizenship and Immigration Services reported credible fear claims for 105,439 migrants in FY 2019 (compared to 22,065 in FY 2018) [2] which is the mechanism for migrants to seek asylum in the US [3]. This increase in the number of migrants in Immigration Law Enforcement (ILE; i.e., CBP and Immigration and Customs Enforcement [ICE]) custody has resulted in increased hospital admissions for migrants under custody in certain states, especially Arizona, California, Texas, and Louisiana [2]. One driving factor behind these increased hospitalizations is CBP’s 1994 policy of prevention through deterrence, which tried to curb border crossing by erecting physical blockades in safer, urban crossing areas that pushed migrants to desert areas that might provide environmental barriers to crossing. The increased number of migrants crossing through harsh desert climates because of this policy has led many to present with severe injuries secondary to falls and environment, dehydration, hypo/hyperthermia, or envenomation [4–6]. The Emergency Medical Treatment and Active Labor Act of 1986 (EMTALA) requires that emergency services receiving Medicare funding treat, triage, and stabilize any person in need regardless of immigration status and ability to pay. Trauma-related injuries, such as falls from the border wall, are often severe enough to require emergent transportation to the trauma center nearest the border [7]. Additionally, many patients present in CBP/ICE custody for obstetrics-related care. With large numbers of migrants now in CBP/ICE detention facilities awaiting asylum and removal court dates for periods of weeks to years [2], patients can present with a wide variety of medical problems related to detention (i.e., infection, mental illness, exacerbation of chronic conditions, etc.) [8, 9] or other underlying general health issues. As such, numerous clinical & hospital services across the southwest border regularly interact with ILE agents and the patients in their custody. The presence of law enforcement (police, sheriffs, corrections officers, etc.) in the clinical setting has been questioned in the past as impeding staff from providing effective care due to damaging the trust between providers and patients and potentially leading to patient privacy breaches [10–12]. However, there is a paucity of evidence on the impacts of ILE specifically and outcomes in the care of patients under immigration custody in the hospital setting [11].

### Current knowledge and gaps

Previous policy work and research have provided limited guidance on the interaction of patients and providers with local law enforcement and corrections officers. While the Health Insurance Portability and Accountability Act of 1996 (HIPAA) allows for the sharing of a patient’s private health information when there are concerns for public safety, the presence and practices of law enforcement officers in hospital settings (i.e., shackling patients, being present for exams, etc.) pose ethical issues for providers in maintaining patient autonomy and beneficence [13–18]. Additionally, no consistent policies exist across medical specialty societies, hospitals, municipalities, states, or the federal government to govern the rights of patients in custody [19].

Even less knowledge exists on how HIPAA, restraint policies, and patient-provider-ILE relationships apply to such patients detained by ILE. While some hospitals have developed policies limiting non-detained patient exposure to ILE in sanctuary hospital settings [20] and some research addresses aspects of care (especially payment) for undocumented immigrants seeking care on their own [6, 21–24], the authors are unaware of any research or consistent policy regarding patients in ILE custody in the hospital setting.

### Objectives of study

This study aimed to determine how the presence of ILE officials affects patient care and provider experiences in a hospital setting. To do so, a pilot survey was designed to answer three specific questions in a single institution study: (1) What experiences do providers have with ILE in caring for detained patients, and how much do providers know about their patients’ rights in this setting? (2) How do providers perceive the respectfulness of ILE toward patients, providers, and other hospital personnel? (3) How frequently do providers perceive potential HIPAA violations in providing care to patients under ILE custody? To answer these questions, providers at all levels of training (i.e., from medical students in undergraduate medical education (UME) to attending physicians) and in all specialties at a Level 1 Trauma Center [25] in Arizona were surveyed on their experiences using both closed-ended and open-ended questions.

Based on anecdotal experience and the lack of guiding policy in this area, it was hypothesized that providers would have knowledge gaps surrounding the rights of patients, and that the presence of ILE in the healthcare setting would threaten both patient privacy and patient care (due to lack of respect). To the authors’ knowledge, this study is the first of its kind which analyzes the impacts of ILE in a hospital setting.

## Methods

### Study setting

This study was a cross-sectional survey of all advanced practice providers (APPs), medical students, residents, fellows, and attending physicians at a Joint Commission accredited teaching hospital system in Arizona. The hospital system comprises two campuses, totaling 765 hospital beds and 31,046 total patient discharges in 2022. The study population was surveyed using probability sampling via a standardized recruitment email distributed using appropriate electronic mailing lists (i.e., each graduating class medical student mailing list and each residency program’s [emergency medicine, family medicine, obstetrics/gynecology, orthopedic surgery, general surgery, psychiatry, internal medicine, and pediatrics] mailing list). The email contained an anonymous, de-identified link to the online Qualtrics survey. Over the course of data collection, each population (260 clinical faculty or APP, 737 residents and fellows and 367 medical students) was sent the initial recruitment email and two standardized reminder emails. A total of 332 (24.3%) responses were collected between June and September 2019.

**Fig. 1:**
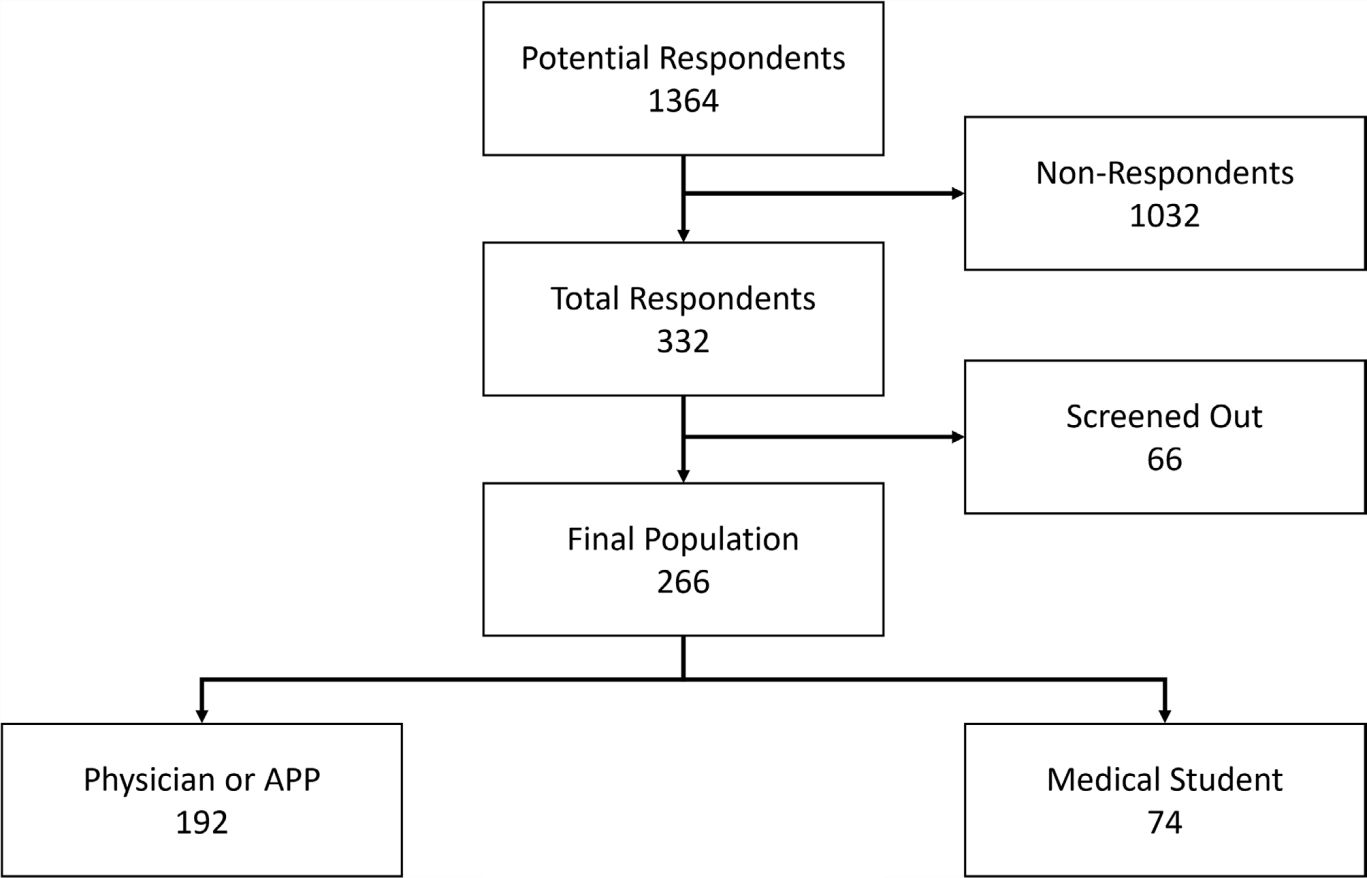
Participant flow diagram

Medical students who completed two or fewer 6-week rotations at the study hospital site, and APPs, residents, fellows, and attending physicians who spent less than 25% of their time providing direct patient care (i.e., all activities related to the provision of care including consultations, as opposed to other tasks such as administrative tasks/billing, research, or teaching) at the study hospital were excluded from the survey. Any respondents who had no interactions with patients in ILE custody were also excluded. All respondents who were excluded were directed to the final demographic questions, and a question that asks about personal familiarity with several topics relating to caring for patients in ILE custody. Respondents who completed less than 45% of the survey were also excluded from the analysis. Thus, 266 responses (79%) were analyzed following initial exclusion (Fig. 1).

### Questionnaire design

The pilot survey (included in the appendix) addressed several topics including the level of experience in caring for patients in ILE custody and the respondents’ perception of respect between ILE officers and the respondent, between ILE officers and the patient in their custody, and between ILE officers and other hospital personnel (e.g., nurse, social worker, medical interpreter, cleaning staff). Respect was defined as being cooperative, providing information with prompting and leaving the room when asked. Each of these questions on perceived respect had a four-point Likert scale ranging from “very respectful” to “somewhat respectful” to “somewhat disrespectful” to “very disrespectful.” Additionally, in each of the above questions, respondents had the opportunity to elaborate by entering free text. Also, the survey assessed perceived violations of patients’ privacy and autonomy in interactions with ILE by determining when ILE was present in the room for patient exams, when they were used as medical interpreters, when they pushed for medical discharge, and when they signed a patient’s forms on their behalf. The survey also contained a series of demographic questions, including age, gender, race/ethnicity, and languages spoken by the respondent with patients in the hospital. Finally, the survey assessed the comfort level of respondents with several topics, such as hospital policies and patient rights, relating to caring for patients in immigration law enforcement custody.

### Ethical considerations

All survey questions, recruitment emails, and data collection methods were in accordance with the Belmont Report, which is itself in line with the Helsinki Declaration of 1964, and were approved by both an academic Institutional Review Board (IRB00000291) and the study hospital’s Institutional Review Board, and informed consent was obtained from survey respondents before they began the survey.

### Quantitative data processing

Results for all responses for each survey population – medical students and physicians – were tabulated and reported as both an absolute number of participants and percentage of respondents in each category. Demographic results were aggregated and tabulated to understand the surveyed population. Likert-scaled responses were categorized as “respectful” (somewhat and very respectful) or “disrespectful” (somewhat and very disrespectful). Likert-scaled responses on the level of comfort with different topics related to ILE presence in the hospital were similarly binarized as “comfortable” (somewhat and very comfortable) or “uncomfortable” (somewhat and very uncomfortable). Differences in responses between physicians and medical students were assessed using chi-square tests. Significance for p-values was set at p < 0.001 given the large number of missing answers for medical students.

### Qualitative data processing

**Fig. 2:**
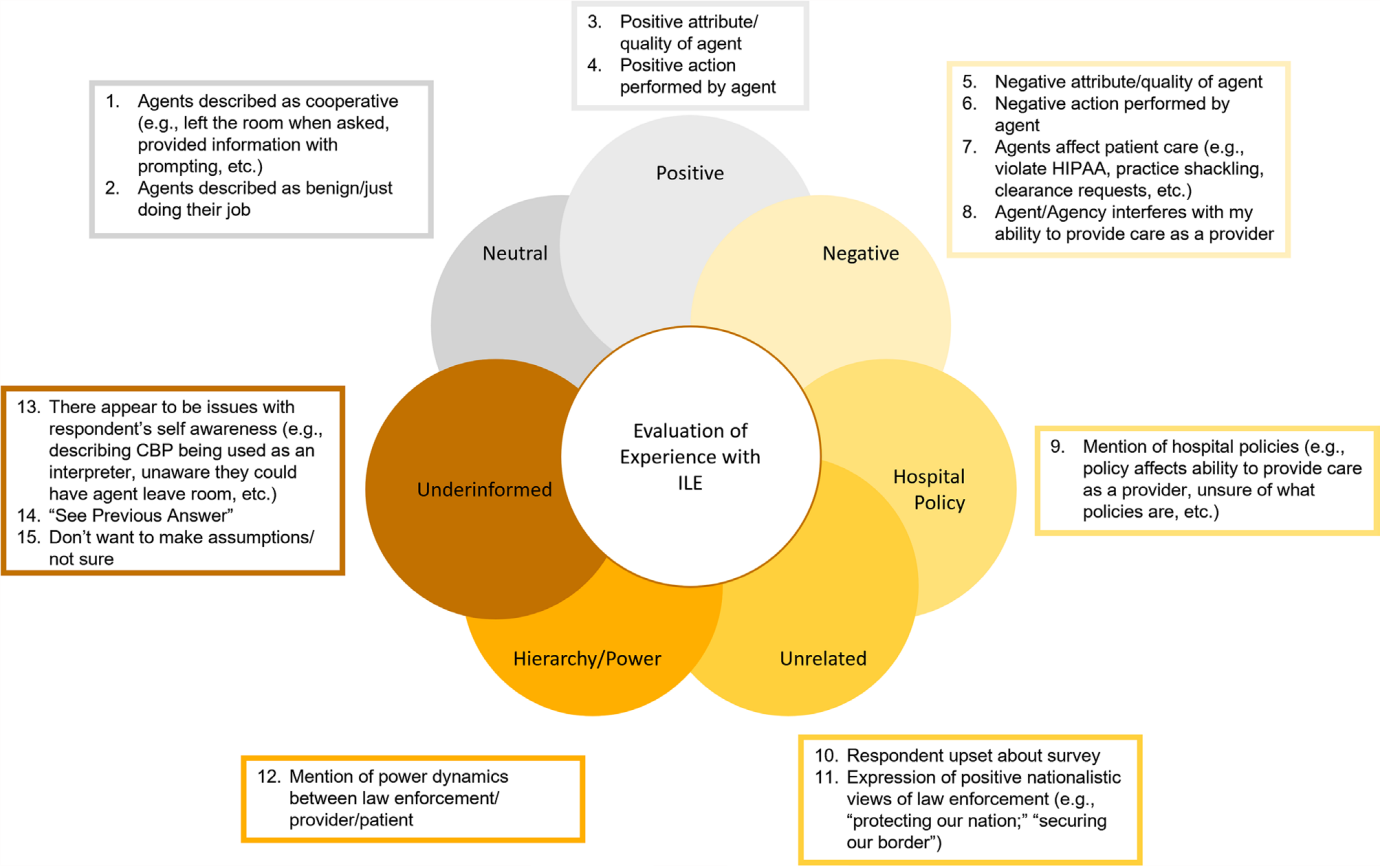
Themes and subthemes used for qualitative analysis of survey comments

Six specific free text comments and one text box for additional end-of-survey free text comments were analyzed for qualitative themes. All free text comments were first analyzed individually by two members of the research team (CL and PL) who constructed subthemes (named as such due to later classification into larger themes) based on comment content. After deliberation by the two researchers, fifteen subthemes were developed which appropriately encompassed all respondents’ comments (see Fig. 2). Following this, four researchers (CL, AA, RR, and CZ) reviewed all comments and assigned one or more of the fifteen subthemes to each comment. Next, themes were created by all research team members to better group the fifteen original subthemes. Subthemes were classified into seven overarching themes as shown in Fig. 2.

### Qualitative data analysis

The number of researcher responses for each theme were then analyzed and counted. If more than four individuals classified a comment as fitting a theme, it was counted. If fewer than two individuals classified a comment as fitting a theme, then it was not counted. All other comments - comments that received between two and four researcher assignments - were reviewed and deliberated by the group until a final decision was made. Finally, the two original researchers (CL and PL) made a second pass through all comments to ensure they were accurately counted and thematically categorized.

To determine the distribution of responses, the total number of comments per theme was determined and the percentage of comments per theme was calculated. Additionally, responses that effectively reflected the sentiments of each theme and/or offered a unique perspective were selected for thematic analysis and further discussion within the paper.

### Techniques to enhance trustworthiness

A neutral reviewer (JA) familiar with the study and expert in qualitative study design evaluated the definition of themes, the classification of responses into subthemes, and the categorization into themes. When the neutral reviewer disagreed with the authors on the classification of the comment, the comment was discarded. Additionally, this neutral reviewer provided analysis and feedback for each of the comments selected per theme to ensure bias was minimized.

## Results

### Participants

Survey respondent characteristics are reported in Table 1.

**Table 1:** Survey Respondent Characteristics

	Physician/APP N = 192 (72.2%)	Medical Student N = 74 (27.8%)	Total N = 266
Age (years)*			
Mean (SD)	37.5 (11.9)	26.9 (2.5)	34.6 (11.3)
**Gender**			
Man (%)	87 (45.3)	27 (36.5)	114 (42.9)
Non-Man (%)	90 (46.9)	38 (51.4)	128 (48.1)
Missing (%)	15 (7.8)	9 (12.2)	24 (9.0)
**Ethnicity**			
Non-Hispanic White (%)	114 (59.4)	28 (37.8)	142 (53.4)
All Other Ethnicities (%)	61 (31.8)	36 (48.6)	97 (36.5)
Missing (%)	17 (8.9)	10 (13.5)	27 (10.2)
**Languages Spoken**			
English Only (%)	76 (39.6)	41 (55.4)	117 (44.0)
Multilingual (%)	102 (53.1)	26 (35.1)	128 (48.1)
Missing (%)	14 (7.3)	7 (9.5)	21 (7.9)
**Detained Patients Under Care in Last Year**			
0 (%)	37 (19.3)	9 (12.2)	46 (17.3)
1–5 (%)	42 (21.9)	23 (31.1)	65 (24.4)
6–10 (%)	44 (22.9)	10 (13.5)	54 (20.3)
11+ (%)	67 (34.9)	1 (1.4)	68 (25.6)
Missing (%)	2 (1.0)	31 (41.9)	33 (12.4)

*Age missing for: 24 physicians & APPs, 12 medical students

Physicians included residents, fellows, and attending physicians.

Amongst the medical student group, 51.4% of respondents identified with a gender other than “man” compared to 50.4% of the general population to whom the survey was distributed; 37.8% of respondents were white compared to 53.8% of all medical students. While demographic data was previously compiled for medical students, similar population data for residents and faculty was not available. Nearly 90% of respondents had interacted with at least one patient in ILE custody in the last year. Among providers, 34.9% had cared for more than eleven detained patients in the last year.

### Survey responses

Approximately 30% of respondents did not answer the questions regarding respectful encounters with ILE. Among those that answered, the majority felt that encounters were respectful (e.g., the ILE officer was cooperative, provided information with prompting, and left the room when asked). Among the disrespectful encounters, most respondents indicated that the disrespect was towards the patient in custody with over 10% of those that responded acknowledging a disrespectful encounter between ILE and patients (Table 2).

**Table 2:** Perceived Respect in Encounters by Group

		Physician/APP N = 192 (72.2%)	Medical Student N = 74 (27.8%)	Total N = 266
Immigration law enforcement & the patient in their custody (%)*	Respectful Encounter	126 (65.6)	21 (28.4)	147 (55.3)
	Disrespectful Encounter	24 (12.5)	13 (17.6)	37 (13.9)
	Missing	42 (21.9)	40 (54.1)	82 (30.8)
Immigration law enforcement & the respondent (%)*	Respectful Encounter	143 (74.5)	31 (41.9)	174 (65.4)
	Disrespectful Encounter	6 (3.1)	1 (1.4)	7 (2.6)
	Missing	43 (22.4)	42 (56.8)	85 (32.0)
Immigration law enforcement & other hospital staff (%)*	Respectful Encounter	139 (72.4)	29 (39.2)	168 (63.2)
	Disrespectful Encounter	7 (3.6)	1 (1.4)	8 (3.0)
	Missing	46 (24.0)	44 (59.5)	90 (33.8)

*Chi-squared test between medical student and physician/APP group responses had p < 0.001

Approximately half of all respondents did not feel confident in their knowledge regarding policies about ILE in the clinical environment. Medical students were the group most likely to respond that they were not confident in their knowledge (Table 3).

**Table 3:** Confidence in knowledge regarding policies related to ILE in the clinical environment:

		Physician/APP N = 192 (72.2%)	Medical Student N = 74 (27.8%)	Total N = 266
1. Hospital policies regarding medical care for detained patients (%)*	Unconfident	78 (40.6)	47 (63.5)	125 (47.0)
	Confident	102 (53.1)	20 (27.0)	122 (45.9)
	Missing	12 (6.3)	7 (9.5)	19 (7.1)
**2. Immigration law enforcement policies regarding medical care for detained patients (%)**	Unconfident	96 (50.0)	45 (60.8)	141 (53.1)
	Confident	84 (43.8)	22 (29.7)	106 (39.8)
	Missing	12 (6.3)	7 (9.5)	19 (7.1)
**3. Level of authority of law enforcement in a medical setting (for example: detaining a person in the hospital, jurisdiction within a hospital) (%)**	Unconfident	90 (46.9)	45 (60.8)	135 (50.8)
	Confident	90 (46.9)	22 (29.7)	112 (42.1)
	Missing	12 (6.3)	7 (9.5)	19 (7.1)
**4. Level to which law enforcement can be involved in patient care (for example: being present during medical exams, filling out patient forms, interpreting) (%)**	Unconfident	82 (42.7)	41 (55.4)	123 (46.2)
	Confident	98 (51.0)	26 (35.1)	124 (46.6)
	Missing	12 (6.3)	7 (9.5)	19 (7.1)
**5. Rights of patients who are in immigration law enforcement (for example: to make a personal phone call, HIPAA protections) (%)**	Unconfident	89 (46.3)	42 (56.8)	131 (49.2)
	Confident	89 (46.3)	25 (33.8)	114 (42.9)
	Missing	14 (7.3)	7 (9.5)	21 (7.9)
**6. Rights of providers of patients in immigration law enforcement (for example: asking an agent to leave a patient’s room, soliciting legal or social services for the patient) (%)**	Unconfident	83 (43.2)	40 (54.1)	123 (46.2)
	Confident	96 (50.0)	27 (36.5)	123 (46.2)
	Missing	13 (6.8)	7 (9.5)	20 (7.5)

*Chi-squared test between medical student and physician/APP group responses had p < 0.001

### Qualitative data coding results

**Fig. 3:**
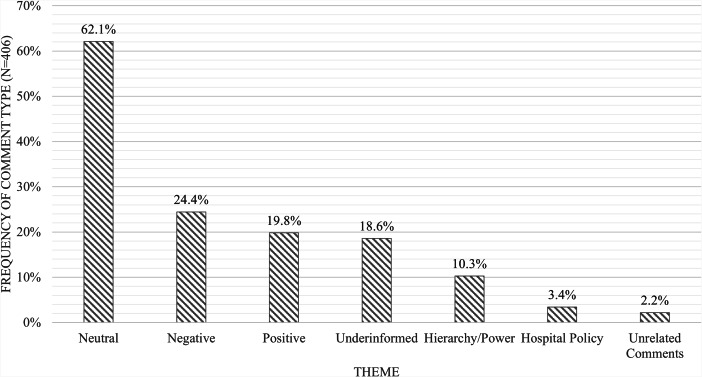
Number of Coded Responses per Theme

Most survey respondents’ comments expressed neutral sentiments toward the presence of ILE in the healthcare setting and toward their specific treatment of patients, providers, and/or personnel (Fig. 3). 24.4% and 19.8% of responses expressed negative and positive sentiments, respectively (Fig. 3), while 18.6% of responses expressed an underinformed opinion - either by failing to acknowledge the rights of patients with law enforcement officials (i.e., the practice of shackling being used) or by failing to recognize the role and scope of ILE in the hospital (i.e., ILE agents being used as medical translators) (Fig. 3).

An additional group of comments (10.3%) mentioned a power dynamic between the ILE agent and the provider, patient, and/or hospital personnel (Fig. 3). Finally, a small percentage of respondents’ comments had issues with the study hospitals’ policies surrounding ILE in the healthcare setting (3.4%) or were unrelated to the questions asked (2.2%).

The specific breakdown of subthematic responses for each theme is shown in Table 4 to further illustrate the vast range of comments and thoughts on ILE presence in the clinical environment. Because each comment could be categorized into multiple subthemes, the total percentage of classified comments exceeded 100%. If a comment fit into two or more sub-themes under the same overarching theme, it was only counted for the overarching theme once.

**Table 4:** Number of Comments per Theme and Subtheme

Overarching Theme/Subtheme	Comments (n = 406)
Neutral (%)	254 (62.1)
1. Agents described as cooperative (i.e.: left the room when asked, provided information with prompting, etc.) (%)	71 (17.4)
2. Agents described as benign/just doing their job/neutral (%)	231 (56.5)
**Positive (%)**	**81 (19.8)**
3. Positive attribute/quality of agent (%)	68 (16.6)
4. Positive action performed by agent (%)	27 (6.6)
**Negative (%)**	**100 (24.4)**
5. Negative attribute/quality of agent (%)	34 (8.3)
6. Negative action performed by agent (%)	46 (11.2)
7. Agents affect patient care (violate HIPAA, practice of shackling, clearance requests, etc.) (%)	71 (17.4)
8. Agent/Agency interferes with my ability to provide care as a provider (%)	31 (7.6)
**Hospital Policy (%)**	**14 (3.4)**
9. Mention of Hospital Policies (they affect my ability to provide care as a provider, unsure of what the policies are, etc.) (%)	14 (3.4)
**Unrelated (%)**	**9 (2.2)**
10. Respondent upset about survey (%)	3 (7.3)
11. Expression of positive [nationalism] views of law enforcement (“protecting our nation, “securing our border”) (%)	8 (2.0)
**Hierarchy & Power Dynamics (%)**	**42 (10.3)**
12. Mention of power dynamics between law enforcement/provider/patient (%)	42 (10.3)
**Underinformed (%)**	**76 (18.6)**
13. There appear to be issues with respondent’s self-awareness (describing CBP being used as interpreter, unaware they could have agent leave room, etc.)	35 (8.5)
14. “See previous answer” (%)	14 (3.4)
15. Don’t want to make assumptions/not sure (%)	27 (6.6)

### Representative responses

Representative responses for each free-text survey item were selected by study authors to demonstrate how responses were codified into themes and to exhibit the range of both specific and generalized experiences with ILE in the healthcare setting. They are reported here as stated by respondents (i.e., with grammatical and other mistakes included) along with a brief explanation of how they were coded by theme and subtheme.

“Why did you think that immigration law enforcement was respectful to the detained patient?“

Response 1: “I think it’s hard to quantify this: because I had several ICE agents that were absolutely cordial, respectful of the women and children in their custody, and respectful of us. Conversely I had several who were absolutely awful to have to deal with, talked smack about “those people” in front of patients, and assumed I - as a white male - have similar views. I think there tend to be agents who grew up around here, or are from similar border communities, and then there are people who get shipped here from parts of the country where they don’t speak spanish, or have our border community identity”.

Response 1 captures three concurrent themes that were reflected in many of the comments the authors analyzed. These themes are neutral (“respectful”), positive (“absolutely cordial”), and negative (“absolutely awful;” “talked smack”) sentiments regarding ILE’s respectfulness with patients. As with many other free-text comments, the respondent answered in general terms about their experience with ILE and not about specific instances. Additionally, rather than highlighting the structural and legal impact of agents in the hospital setting, this respondent chose instead to focus on the summative experience of all their interpersonal interactions with agents. In addition to expressing general sentiments that run the gamut, this respondent also brings up elements of relatability and understanding, deemed necessary to a positive experience with ILE. According to the respondent, those agents that “speak spanish” and “have our border community identity” might be more respectful to patients and healthcare personnel.

Response 2: “They helped with interpretation as needed, didn’t try to force is to discharge patient. Wanted to ensure medical clearance for discharge”.

Response 2 captures three concurrent themes as well - neutral, positive, and underinformed. The last of these is especially poignant. Underinformed responses are those that do not recognize the clearly established rights of patients in this setting. This quote captured a specific practice that is oppositional to standard ethical expectations (often enforced by hospital policies and/or local/federal law) that indicates the respondent is underinformed. As interpreters are specifically licensed and employed to translate in a healthcare setting, the use of family members, friends, or ILE agents as interpreters is often considered ethically inappropriate. As such, the fact that this respondent included “they helped with interpretation as needed” indicates that ILE agents were inappropriately used as interpreters and calls into question the training that this provider has had regarding interpretation services and the ethics behind it. Such underinformed comments were common across many respondents.

Response 3: “They were courteous to the patient and allowed for them to receive the appropriate care that they were brought to the emergency department. Often times, they would bring up concerns that were not the intended reason for transfer to the hospital. Last night, I had a patient that was sent for medication refill but the BP agent voiced a concern that the patient had not urinated for greater than 24hours and went over 36hours without food or water”.

Response 3 captures a specific event that has both neutral and positive themes. While codifying various responses, those that mentioned courtesy or professionalism were commonly coded as neutral (as these are expected neutral practices within the ILE profession and their interactions with healthcare providers). As such, when the respondent proffers that the agents were “courteous to the patient,” they reflect neutrally on their behavior toward patients. Additionally, this quote highlights both a generalized positive statement about the fact that ILE agents mentioned concerns about patients and a specific example of this statement in which an ILE agent showed extra concern beyond what was expected of them in their professional duties to ensure that a patient was adequately fed and hydrated.

“Why did you think that immigration law enforcement was disrespectful to the detained patient?“

Response 4: “While in the Emergency Room we were ruling out a heart attack on a detained patient while two border patrol were in the room. They saw my classmate and I were medical students and they started lecturing us on how the patient was ‘playing us’ and the he was making it all up to get out of the detention center. Then they proceeded to say we should not forget to check for parasites by saying; ‘if you’re dealing with central americans you always got to check for parasites, sometimes with the Mexicans too.’ I felt compelled to speak up but wanted to focus on my patient and did not want my patient to have repercussions if I were to confront the Border Patrol officer’s lack of empathy/respect for my patient.”

Response 4 represents both negative and hierarchical/power related themes. The negative sentiments of the response come from a specific interaction with ILE agents in which they used language that could be considered harmful, especially regarding stereotypes of Central American and Mexican migrants and regarding migrants’ alleged abuse of the system. The response also highlights a specific power dynamic between ILE and medical students. The respondent outlines how the medical student felt powerless to confront the ILE agent about their negative statements out of fear for negatively affecting their patient’s care.

Response 5: “CBP have some agents that have been acclimated to an institutional culture of crassness and dehumanization. One BP agent, who spoke no Spanish, said things like “75% of them don’t make their trial”, and “How could you put your kid in that much danger?” Without realizing that I volunteer my time with migrants at a local Shelter and actively despise CBPs presence in a children’s hospital. [Study site] needs a common sense policy to handle their presence. It’s contrary to a place of healing for children in need.”

Response 5 highlights negative and hospital policy related themes. The negative theme is found in the generalized sentiment regarding CBP’s “culture of crassness and dehumanization.” The respondent also explicitly responds that the hospital study site needs “a common sense policy” regarding immigration law enforcement. Lack of clarity in the policies at the study site (and in general local and federal policies) was stated in multiple quotes.

“Why did you think that immigration law enforcement was respectful to you [the provider]?“

Response 6: “I’m a doctor in a white coat.”

Response 6 exhibits an inverse power dynamic than that stated in Response 4. By highlighting their position as a physician in a white coat, the respondent seems to show that they deserve respect due to the hierarchical nature of their position in the hospital setting.

Response 7: “The majority of officers have not impeded my ability to deliver care. They have been cordial. I have never asked one of them to leave a room because I never did anything that required them to leave, so I am judging respect based off of good interactions.”

Response 7 ascribes to neutral, negative, and underinformed themes. In the neutral light, the description of the officers as “cordial” and “respect[ful]” represents expectations of ILE’s professional duty to be courteous with the individuals they interact with. The negative theme was so assigned because of omission. That is to say that this author specifically mentions that “the majority have not impeded my ability to deliver care.” Indicating this majority, also indicates that there were a minority of ILE agents that *did* impede care, which fits into the negative overarching theme. Finally, the underinformed theme was found in the respondent’s assertion that they did not have to ask the agents to leave the room. As there are specific scenarios in which HIPAA would require a private interaction with a patient, this assertion demonstrates that the respondent was underinformed in protecting their patients’ right to privacy.

“Why did you think that immigration law enforcement was respectful to hospital personnel?“

Response 8: “I feel like it’s hit or miss. Some are very nice and respectful while others can be belligerent more so to nursing staff etc.”

Response 8 captured both neutral and negative sentiments in a generalized manner. The description of ILE as “nice and respectful” neutrally states their ability to act professionally in the hospital setting. However, the “miss” and “belligerent” behavior indicate that ILE’s interaction with other hospital staff can have negativity attached.

“In the space below, please provide additional experience you have with immigration law enforcement at BUMC:“

Response 9: “Our LAW ENFORCEMENT officials that I have interacted with in the [Emergency Department (ED)] have been nice to work with and respectful. They have never given me push back when I asked them to leave for a sensitive exam or when I was asking sensitive questions. These immigrants are in their custody because they broke laws. Because of this, our law enforcement officials are obligated to keep them detained and ensure they do not escape. In my opinion they could be much more strict (shackles, restrictions, etc) but they do not. I have never had one of them be disrespectful to a patient. I have never had them be abusive or demeaning. They follow orders and regulations just like any other law enforcement agency who deals with individuals who break laws. I appreciate that they do a good job of enforcing the law at our borders.”

This quote was codified as possessing unrelated, underinformed, neutral, and policy themes. The unrelated theme was codified due to the last sentence of the comment, which discussed appreciation for the work that ILE agents did at the border, a statement that was unrelated to the purpose of the survey which was addressing the interactions of ILE, patients, and staff in the medical setting. The underinformed theme was highlighted as the respondent discussed the need for more strict regulations on patients that misalign with standard protections for patients under any law enforcement authority and against patient privacy and autonomy in certain situations. The neutral theme was again selected as agents were “not disrespectful, “never…abusive or demeaning” and as “[t]hey follow orders and regulations,” indicating that ILE officers were neutrally conducting their roles in the hospital setting. The policy theme was chosen for this comment as it discusses changes that could be made to allow the officers to be “more strict.”

Response 10: “They rush patients in the shower and threaten to come in if they don’t hurry. They have watched a mother breastfeed her daughter (patient). They have made jokes about the patient when immigration news was on the television. border patrol agents kept asking for updated medical information on patients and when they would be discharged.”

This quote was codified as possessing negative and hierarchy/power themes. The negative theme comes from three specific negative actions that the respondent witnessed – the first having to do with rushing a patient in a medically appropriate and necessary action related to personal hygiene; the second had to do with inappropriately disrespecting a patient’s privacy and dignity while she breastfed, and the third had to do with targeted comments against immigrants. The comment was also codified as one which discusses hierarchy and power as the respondent seems to point out how ILE agents constantly use their role and power against patients and in ways that rush providers.

Response 11: “Medical students, providers, and staff need training on the fact that HIPPA still applies to detained patients!!!!!!!!!!!!! I after I was finishing up doing seeing my patient in the morning, the border patrol agent in the room asked me how many more days would the patient need to be here. I had told the patient he had some labs that were downtrending but still way to elevated to be discharge, so the border patrol agent must have spoken some Spanish because he then ask me what labs are still elevated that are keeping the patient here. I responded some kidney labs. He said which ones. I then said that I didn’t not believe that I could share patient information with him. He replied that according to his understand, HIPPA did not apply to him and that I could tell him patient information. I respectfully responded that that was contrary to my understand and I would need to check with my attending to clarify. The only reason I felt confident to give that response if because I had just attended a talk that same week put on by the [study site] residents/attendings) on what our rights are as providers with respect to patients in Border Patrol/ICE/etc custody!!!!! EDUCATE ALL WITH INTERACTION WITH PATIENTS PLEASE !!! Also people should be encouraged to ask the officers to step outside of the room during patient interactions.”

This quote was codified as possessing negative and policy themes. The negative part of the comment points out a specific action that an ILE agent took to attempt to coerce a trainee into discussing protected health information pertaining to a patient. The second highlights the need for education and training as the respondent multiple times with emphatic language and exclamation marks, proclaims that providers at the study site need more clear training on the policies and the rights that they have in protecting migrant patient information.

Response 12: “Having immigration law enforcement around is often an impediment to quality care. They should not be allowed in the operating room and should be held to HIPAA regulations as are all of us.”

This quote was codified as possessing negative and policy themes. The respondent first gives a general statement that highlights a potential negative effect of ILE presence on patient care while the second part of the comment requests a change in policy that might better protect patient privacy in the operating room (as well as in other areas of the hospital).

## Discussion

There has long been debate regarding patient rights and law enforcement interactions in the hospital setting, with various groups, including the American College of Emergency Physicians and the American Medical Association [26, 27], advocating for patient rights and interests being preserved by physicians and institutions. These study results are the first of their kind, with no other data available specifically examining this topic. The results solidify that refined, uniform, and specific policies should be directed at prioritizing the rights of patients. HIPAA prohibits release of information to law enforcement without patient consent [28]; however, policies must be examined and revised to determine that institutions are not giving “carte blanche” access to patient information in their security policies, thus breaching HIPAA, violating patient rights, and emboldening ILE agencies to further encroach on constitutional rights and cause potentially disrespectful encounters. Indeed, the need for these clear, specific policies is highlighted in our study by the mention of policies in 3.4% of comments and the 18.6% of comments that were underinformed specifically around what ILE agents can and cannot do in the clinical environment. The representative responses provided in the comment analysis above further bolster the need for clarity in this realm.

This topic is one that is not common in medical training and attempts are presently being made at addressing and integrating these medicolegal ethics into medical education and continuing education curricula to bridge the gap for students, trainees, and faculty. Approximately half of survey respondents in this study reported they were not confident in knowing their rights as providers, the rights of patients, and the policies in place for patients in custody, indicating that physicians and medical trainees would benefit from relevant education. This education would include reading and lectures on immigration policies as well as important statistics like the proportion of migrants in ILE custody that are deported. Such a framework might help trainees and providers better understand the motivation behind relevant guidelines for interactions with ILE. Additionally, as can be seen in the disparate number of perceived respectful encounters between students and physicians, there is a clear opportunity to provide more training to medical students in early stages about interacting with ILE. As such, we would strongly recommend the development of educational materials for medical trainees and physicians throughout the training pipeline to ensure that they can navigate these interactions in a way that is ethical and provides patients with the greatest level of care possible.

The study survey defined disrespectful encounters as “negative attribute, qualities, actions of agent and agents affecting patient care through violating HIPAA, practice of shackling, clearance requests, and agent/agency interferes with my ability to provide care as a provider.” Although most encounters did not include such disrespect, the presence of any of these encounters may be a detriment to quality of care that is provided by hospitals and health systems as previously described in the literature [29]. Additionally, thematic analysis of comments made by survey participants revealed that nearly a quarter of comments had negative themes within them, including negative attributes and actions of agents and negative effects of agents on patient care. As such, policies must be revised to prevent these types of encounters while allowing medical personnel to adequately ensure patient rights and safety. Previous studies have determined that immigration deterrent policies and rhetoric aimed at immigrants are a major determinant of health, leading to worse outcomes as well as increased screening for depression, anxiety, and post-traumatic stress disorder (PTSD) [30].

Immigration detention has been an issue following the implementation of policies that were enforced by the Clinton, Bush, and Obama administrations. With increased and more severe policy enforcement, separation of minors as a deterrence tactic, visibility of poor detention camp conditions in the polarizing period of the Trump administration [31–33], hospitals have been unprepared for the challenge of providing ethical and consistent care that respects the basic rights of patients held in detention by immigration authorities. This current landscape leads hospital groups and medical professionals that are contracted with ICE, CBP, and law enforcement agencies to grapple with the reality that comes with taking care of patients in custody.

### Limitations

The survey was prone to non-response selection bias; it was likely that the respondents who had strong feelings about the survey topic were more likely to respond compared to those in the sampling frame with indifference or mild feelings towards the topic. Another limitation was the fact that there were likely respondents who were afraid to respond to a survey that centers on a sensitive topic. To mitigate this, it was emphasized that all responses were to remain anonymous. Although keeping this survey anonymous required sending out reminder emails to all the target population, it was critical to protect respondents’ privacy to alleviate potential non-response from fear of professional or personal repercussions. Moreover, this anonymity also meant it was possible that individuals filled out the survey more than once.

Another limitation of this project was the response rate calculation. Because the survey link was sent electronically via email, it is difficult to know how many individuals opened the recruitment email. For this reason, the actual response rate may be higher than the calculated response rate of 24.3% if we were able to account for only the individuals who opened the survey email. Another challenge of this project was recall bias, as respondents may forget and/or omit details of their experiences, thus affecting survey results.

An additional limitation of the project was the number of missing responses for the medical student group, which had a pronounced effect on the p-values in Table 2. Because of these missing answers, it was difficult to fully interpret how differently medical students and providers perceive the respectfulness of immigration law enforcement in the clinical setting.

This study focused on the perspectives of medical students, residents, fellows and attending physicians. It did not capture numerous other actors within the hospital, such as nurses, medical interpreters, and social workers. However, each of these actors would also have valuable experiences relating to interactions with patients in immigration law enforcement custody. Moreover, the perspectives and experiences of the actual patients in immigration law enforcement custody, as well as their family members, were not reflected in this study. This is an important limitation to note, as some of the questions asked in the survey, such as a respondents’ perception of respect between ILE and patient in custody, are once removed from the actual feeling of respect and disrespect experienced by the patient. Looking forward, it would be valuable to capture these perspectives in future surveys, as they may help to provide a more complete and thorough landscape of the impact of immigration law enforcement on patient care.

## Conclusion

This study is the first of its kind to examine immigration law enforcement impacts on tertiary hospitals in US-Mexico border regions, and future studies may be reproduced in other locales enduring similar circumstances. Thus, this study could serve as groundwork for developing hospital policies to address acceptable actions and interactions of ILE in patient care, for developing legal precedent for immigration detainees in hospital settings, and for developing educational resources to address gaps in provider knowledge regarding the rights of patients under the custody of ILE. Additionally, as this is a preliminary study, future ones should further expand upon this survey to look at patients apprehended for deportation and in detention facilities and examining attitudes of patients in custody. As this topic continues to entrench the United States, the authors encourage more studies in this topic as well as timely and comprehensive policy overhaul to allow for ethical care.

### Electronic supplementary material

Below is the link to the electronic supplementary material.

Supplementary Material 1

## Data Availability

The datasets generated and/or analyzed during the current study are not publicly available due to the desire to protect respondent privacy. Because this was single-center study, the authors wanted to prevent identification of any respondents.

## Abbreviations

APP: Advanced Practice Provider
ILE: Immigration Law Enforcement consisting of US Customs and Border Protection and Immigration and Customs Enforcement
CBP: US Customs and Border Protection
ED: Emergency Department
EMTALA: Emergency Medical Treatment and Labor Act
FY: Fiscal Year
ICE: US Immigration and Customs Enforcement
IRB: Institutional Review Board
PTSD: Post-Traumatic Stress Disorder
UME: Undergraduate Medical Education
US: United States
