# Supplementary material for: Assessment of immigration law enforcement presence in a teaching hospital along the US/Mexico border
Source: Int J Equity Health. 2023 Sep 28;22:199. doi: 10.1186/s12939-023-01934-2 (PMC10540385; doi:10.1186/s12939-023-01934-2)
Supplement: Supplementary file 1 — Supplementary Material 1 [file 12939_2023_1934_MOESM1_ESM.pdf]

CBP/ICE in Hospital Survey

Survey Flow

Block: Default Question Block (30 Questions)
Standard: Block 2 (2 Questions)
Standard: Block 1 (1 Question)

Page Break

Claire Lamneck, M.D., M.P.H, University of Arizona College of Medicine – Tucson

Alexander Alvarez, B.S., University of Arizona College of Medicine – Tucson

Cazandra Zaragoza, M.D., M.P.H., University of Arizona College of Medicine – Tucson

Rombod Rahimian, M.D., M.P.H., University of Arizona College of Medicine – Tucson

Mario Trejo, M.P.H., University of Arizona Mel & Enid Zuckerman College of Public Health

Patricia Lebensohn, M.D., University of Arizona College of Medicine – Tucson

Start of Block: Default Question Block

Q1 ***Study Site*** **Consent to Participate in Research** Survey **Study Title:** Practicioner - Immigration Law
Principal Investigator: Patricia Lebensohn, MD

You are being asked to participate in a research study. Your participation in this research study is voluntary and you do not have to participate. This document contains important information about this study and what to expect if you decide to participate. Please consider the information carefully. Feel free to ask questions before making your decision whether or not to participate. The purpose of this study is to ask practitioners about current care and privacy of detained and undocumented patients. Requirements of the study include taking a short anonymous survey, estimate 5 minutes. There are no expected risks to you as a result of participating in this study. You will not benefit directly from participating in this study. If you provide identifying information, your name will not be used in any report. Identifiable research data will be encrypted and password protected. Your responses will be assigned a code number. The list connecting your name to this code will be kept in an encrypted and password protected file. Only the research team will have access to the file. When the study is completed and the data have been analyzed, the list will be destroyed. The information that you provide in the study will be handled confidentially. However, there may be circumstances where this information must be released or shared as required by law. The *Study Site* Institutional Review Board may review the research records for monitoring purposes. For questions, concerns, or complaints about the study you may contact ***Patricia Lebensohn, MD at ***-***-*******. For questions about your rights as a participant in this study or to discuss other study-related concerns or complaints with someone who is not part of the research team, you may contact the Human Subjects Protection Program at ***-***-**** or online at *study site website*. **Signing the consent form**

I have read (or someone has read to me) this form, and I am aware that I am being asked to participate in a research study. I have had the opportunity to ask questions and have had them answered to my satisfaction. I voluntarily agree to participate in this study. I am not giving up any legal rights by signing this form. I will be given a copy of this form (attached to this question)**.** ***By clicking here I agree to participate:***

-

○ Yes (1)

○ No (2)

*Skip To: Q2 If *Study Site* Consent to Participate in Research Study Title: Practicioner Survey - Imm... = Yes*

*Skip To: End of Survey If *Study Site* Consent to Participate in Research   Study Title: Practicioner Survey - Imm... = No*

Q2 Please answer the following questions to the best of your ability. This survey is to remain completely anonymous. Responses will be used to create a report for *study site* Administrators aimed at assessing the current care of detained and undocumented patients at *study site*

When answering the questions below please speak to your experiences from *study site campuses*.

Note: A detained patient, or a patient in detention, refers to any patient in immigration law enforcement custody. Immigration law enforcement refers to any Border Patrol (BP), Department of Homeland Security (DHS) or Immigration and Customs Enforcement (ICE) official.

You may skip any questions you do not wish to answer. Thank you for your time and thoughtfulness.

Q3 Which title best describes you?

- ◯ Medical Student (1)
- ◯ Medical Resident (2)
- ◯ Medical Fellow (3)
- ◯ Physician (4)
- ◯ Other (5) ____

Skip To: Q4 If Which title best describes you? = Medical Student

Skip To: Q6 If Which title best describes you? != Medical Student

Q4 What year medical student are you?

- MS1 (1)
- MS2 (2)
- MS3 (3)
- MS4 (4)
- Other, please specify (5) ____

Q5 In total how many 6-week rotations at a *study* site have you completed?

- 1 or fewer rotations (1)
- 2 rotations (2)
- 3 rotations (3)
- 4 or more rotations (4)

*Skip To: Q25 If In total how many 6-week rotations at a *study* site have you completed? = 1 or fewer rotations*

*Skip To: Q8 If In total how many 6-week rotations at a *study* site have you completed? != 1 or fewer rotations*

Q6 Which of the following is your specialty?

- ○ Family Medicine (1)
- ○ Surgery (2)
- ○ OB/GYN (3)
- ○ Internal Medicine (4)
- ○ Emergency Medicine (5)
- ○ Psychiatry (6)
- ○ Pediatrics (7)
- ○ Other, please specify (8) ____

Q7 In an average week, approximately what percent of your time do you spend on direct patient care* at a *study* affiliated site?
*Patient care, in this case, means all activities directly related to the provision of care including consultations as opposed to other tasks such as administrative tasks/billing, research or teaching

- ○ (1)
- ○ 25-49% (2)
- ○ 50-74% (3)
- ○ >75% (4)

Q8 In the last year, about how many detained patients* have you cared for?
**A detained patient, or a patient in detention, refers to any patient in immigration law enforcement custody*

- 0 patients (1)
- 1-5 patients (2)
- 6-10 patients (3)
- 11+ patients (4)

Skip To: Q25 If In the last year, about how many detained patients have you cared for? *A detained patient, or a... = 0 patients*

Q9 On average, how many times a month do you interact with a patient for any reason (including clinical visits, medical rounding, discharge process, etc.) while immigration law enforcement is present?

- <1 time (1)
- 2-4 times (2)
- 5+ times (3)

Q10 To the best of your knowledge, please select which type of immigration law enforcement officials you have interacted with at *study site* in the last year (check all that apply):

- ☐ Immigration Customs Enforcement (ICE) agent (1)
- ☐ Customs and Border Patrol (CBP) agent (2)
- ☐ Enforcement and Removal Operations (ERO) agent (3)
- ☐ Homeland Security Investigators (HSI) agent (4)
- ☐ Other type of immigration officer: (5) ____

Q11 *Now, think back to the interactions you have had with detained patients in the last year for any reason (such as clinical visits, medical rounds, discharge, etc.)*

Q12 Of all the detained patients you have interacted with in the last year, how frequent have the following situations occurred?

	None of the detained patients (1)	Less than half of the detained patientsi (2)	More than half of the detained patients (3)	All of the detained patients (4)	N/A (5)
Immigration law enforcement was present during personal medical questioning of a minor ((1)	○	○	○	○	○
Immigration law enforcement was present during personal medical questioning of an adult (18 or older) in their custody (2)	○	○	○	○	○

Q13

	None of the detained patients (1)	Less than half of the detained patients (2)	More than half of the detained patients (3)	All of the detained patients (4)
Immigration law enforcement did not leave the patient's room when asked (3)	○	○	○	○
Immigration law enforcement left the room when private medical information was discussed with the patient (4)	○	○	○	○
Immigration law enforcement was used as an interpreter (5)	○	○	○	○
Immigration law enforcement did personal activities in the patient's room (like watching television or taking a phone call) (6)	○	○	○	○
Immigration law enforcement urging medical providers to discharge the patient (7)	○	○	○	○
Immigration law enforcement urging the patient to refuse treatment and/or medication (8)	○	○	○	○
Immigration law enforcement reviewed the patient's medical records (9)	○	○	○	○

Immigration law enforcement filled out medical forms on the patient's behalf (10)	○	○	○	○
A detained patient was discharged to immigration law enforcement without a clear discharge plan (11)	○	○	○	○
Immigration law enforcement asked for a letter that medically cleared them for release to a detention center (12)	○	○	○	○

Page Break

Q14 *Continue to think back to your interactions with immigration law enforcement at *study site* within the* *last year* *and answer the following questions*

Q15 On average, how respectful did you feel that immigration law enforcement was to the patient in their custody?

- ○ Very Respectful (1)
- ○ Somewhat Respectful (2)
- ○ Somewhat Disrespectful (3)
- ○ Very Disrespectful (4)

Display This Question:

If On average, how respectful did you feel that immigration law enforcement was to the patient in th... = Very Respectful

Or On average, how respectful did you feel that immigration law enforcement was to the patient in th... = Somewhat Respectful

Q16 Why did you think that immigration law enforcement was respectful to the detained patient?

____

____

____

____

____

Display This Question:

If On average, how respectful did you feel that immigration law enforcement was to the patient in th... = Somewhat Disrespectful

Or On average, how respectful did you feel that immigration law enforcement was to the patient in th... = Very Disrespectful

Q17 Why did you think that immigration law enforcement was disrespectful to the detained patient?

____

____

____

____

____

Page Break

Q18 On average, how respectful did you feel that immigration law enforcement was to you?

- ◯ Very Respectful (1)
- ◯ Somewhat Respectful (2)
- ◯ Somewhat Disrespectful (3)
- ◯ Very Disrespectful (4)

Display This Question:

If On average, how respectful did you feel that immigration law enforcement was to you? = Very Respectful

Or On average, how respectful did you feel that immigration law enforcement was to you? = Somewhat Respectful

Q19 Why did you think that immigration law enforcement was respectful to you?

____

____

____

____

____

Display This Question:

If On average, how respectful did you feel that immigration law enforcement was to you? = Somewhat Disrespectful

Or On average, how respectful did you feel that immigration law enforcement was to you? = Very Disrespectful

Q20 Why did you think that immigration law enforcement was disrespectful to you?

____

____

____

Page Break

Q21 On average, how respectful did you feel that immigration law enforcement was to other hospital personnel (nurse, social worker, medical interpreter, cleaning staff, etc.)?

- ◯ Very Respectful (1)
- ◯ Somewhat Respectful (2)
- ◯ Somewhat Disrespectful (3)
- ◯ Very Disrespectful (4)

Display This Question:

If On average, how respectful did you feel that immigration law enforcement was to other hospital pe... = Very Respectful

Or On average, how respectful did you feel that immigration law enforcement was to other hospital pe... = Somewhat Respectful

Q22 Why did you think that immigration law enforcement was respectful to hospital personnel?

____

____

____

____

____

Display This Question:

If On average, how respectful did you feel that immigration law enforcement was to other hospital pe... = Somewhat Disrespectful

Or On average, how respectful did you feel that immigration law enforcement was to other hospital pe... = Very Disrespectful

Q23 Why did you think that immigration law enforcement was disrespectful to hospital personnel?

____

____

Page Break

Q24 In the space below, please provide additional experience you have with immigration law enforcement at *study site*:

____

____

____

____

____

Page Break

Q25 How confident do you feel about your knowledge on the following topics?

	Not at all Confident (1)	Slightly Confident (2)	Fairly Confident (3)	Very Confident (4)
Study site's hospital policies regarding medical care for detained patients (1)	○	○	○	○
Immigration law enforcement policies regarding medical care for detained patients (2)	○	○	○	○
Level of authority of law enforcement in a medical setting (for example: detaining a person in the hospital, jurisdiction within a hospital) (3)	○	○	○	○
Level to which law enforcement can be involved in patient care (for example: being present during medical exams, filling out patient forms, interpreting) (4)	○	○	○	○
Rights of patients who are in immigration law enforcement (for example: to a personal phone call, HIPAA protections) (5)	○	○	○	○

Rights of providers of patients in immigration law enforcement (for example: asking an agent to leave a patient's room, soliciting legal or social services for the patient) (6)	○	○	○	○

Page Break

Q26 What is your age?

____

Q27 Which gender best describes you? (check any that apply)

- ☐ Woman (1)
- ☐ Man (2)
- ☐ Transgender (3)
- ☐ Gender Nonbinary (4)
- ☐ Prefer to self-describe (5) ____

Q28 Which race/ethnicity best describes you? (check all that apply)

- ☐ American Indian/Alaska Native (1)
- ☐ Asian (2)
- ☐ Native Hawaiian/Pacific Islander (3)
- ☐ Hispanic or Latino (4)
- ☐ Black or African American (5)
- ☐ White (6)
- ☐ Other (7) ____

Q29 Do you speak any other language(s) besides English with patients at *study site*?

○ Yes (1)

○ No (2)

*Skip To: End of Block If Do you speak any other language(s) besides English with patients at *study site*? No*

Display This Question:

*If Do you speak any other language(s) besides English with patients at *study site*? = Yes*

Q30 Which language(s) do you speak with patients at *study site*? (check all that apply)

▢ Spanish (1)

▢ Vietnamese (2)

▢ Chinese (Mandarin, Cantonese, or other Chinese language) (3)

▢ German (4)

▢ French (5)

▢ Korean (6)

▢ Arabic (7)

▢ Portuguese (8)

▢ Haitian Creole (9)

▢ Other, please specify: (10) ____

End of Block: Default Question Block

Start of Block: Block 2

Q31

We are very interested in your perspectives and would like to learn more about your experiences regarding caring for patients in immigration law enforcement custody. If you are willing to be contacted for a follow-up qualitative interview, please provide your contact information below. Note: If you put your contact information below, your responses will still be confidential and will not be linked to any personal identifiers in analyses or presentations of these data.

Q32 If you agree to being contacted for follow-up, please provide your contact information below:

○ Full Name (1) ____

○ Email Address (2) ____

End of Block: Block 2

Start of Block: Block 1

Q33 Thank you so much for taking the time to respond to this survey. We know how busy you are and greatly appreciate it.

End of Block: Block 1
